# Supplementary material for: Epidemiological Studies of Children’s Gut Microbiota: Validation of Sample Collection and Storage Methods and Microbiota Analysis of Toddlers’ Feces Collected from Diapers
Source: Nutrients. 2022 Aug 12;14(16):3315. doi: 10.3390/nu14163315 (PMC9416069; doi:10.3390/nu14163315)
Supplement: Supplementary file 1 [file nutrients-14-03315-s001.zip › nutrients-1777811-supplementary/nutrients-1777811-supplementary-figure.pdf]

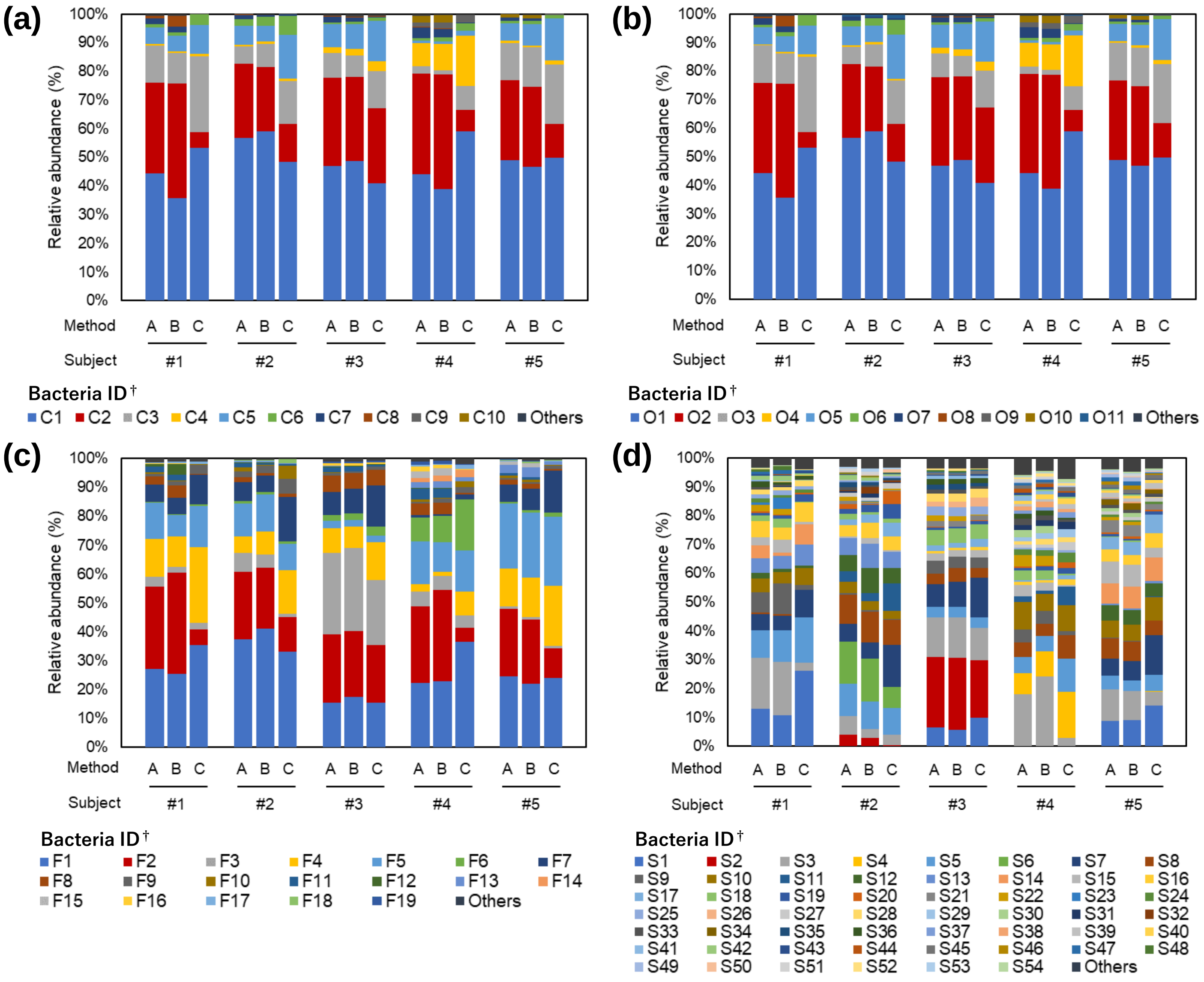

Figure S1. Research I: Fecal bacterial composition at the class, order, family, and species level in five adult volunteers. Bacterial composition at the (a) class, (b) order, (c) family, and (d) species levels.

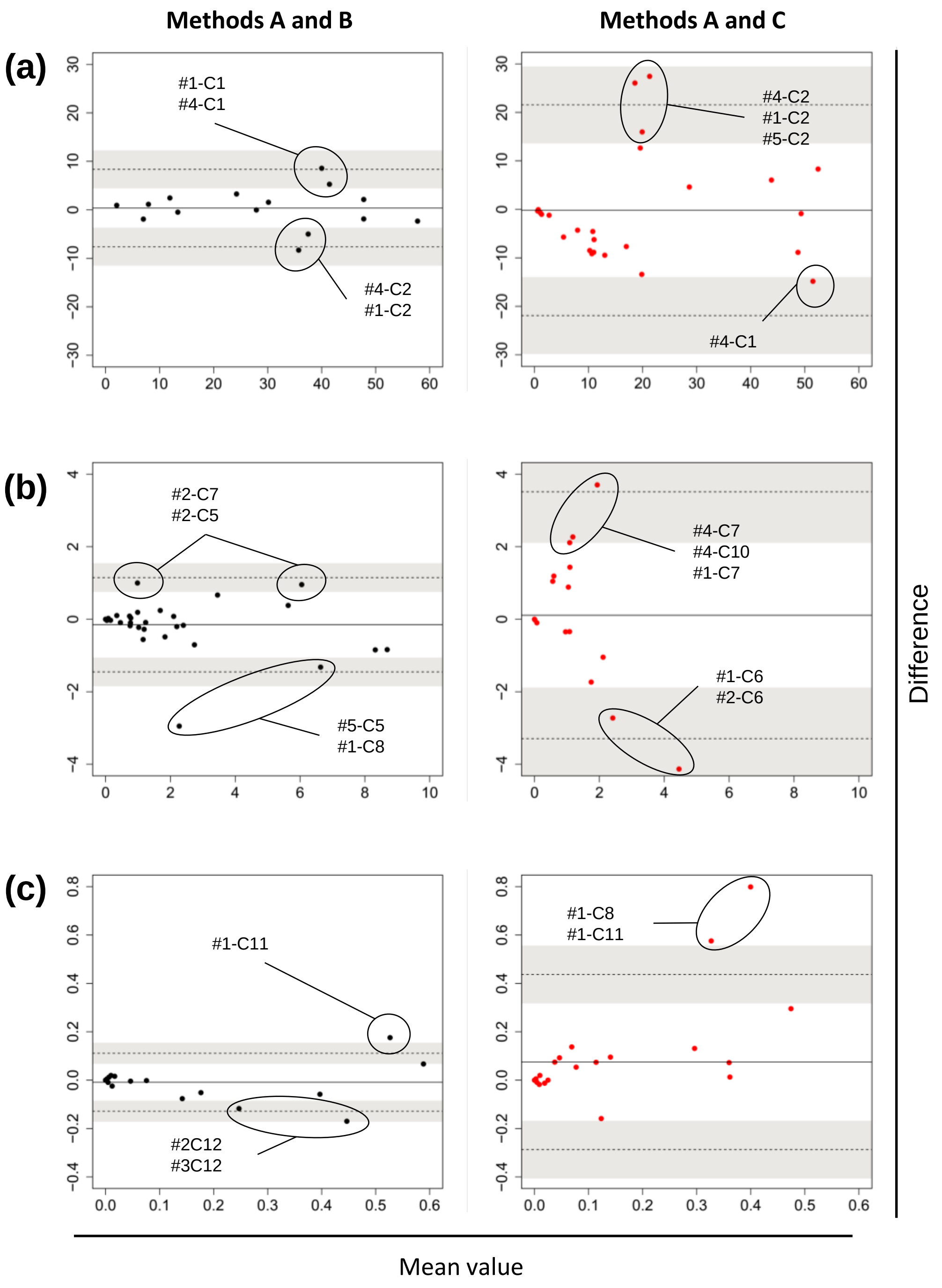

Figure S2. Research Ⅰ: Bland-Altman plots of the relative abundance of fecal bacteria at the class level obtained by each method from five adult volunteers. Comparisons between Methods A–B (left) and Methods A–C (right). The relative abundance of bacteria in each specimen was plotted. (a) Their maximum abundance in any of the specimens was ≥ 10%; (b) ≥ 1% and < 10%; (c) < 1%. Solid lines indicate the means of the differences between the two test values, dotted lines indicate the limits of agreement (the mean of the difference ± 1.96 × standard deviation), and the gray shaded area indicate the 95% confidence interval of the limits of agreement. For Methods A and B, stool samples were collected by commercial collection tubes. For Method C, urine and stool samples were applied to disposable diapers. For Methods B and C, the samples were stored at -80 ℃ for 2 months.

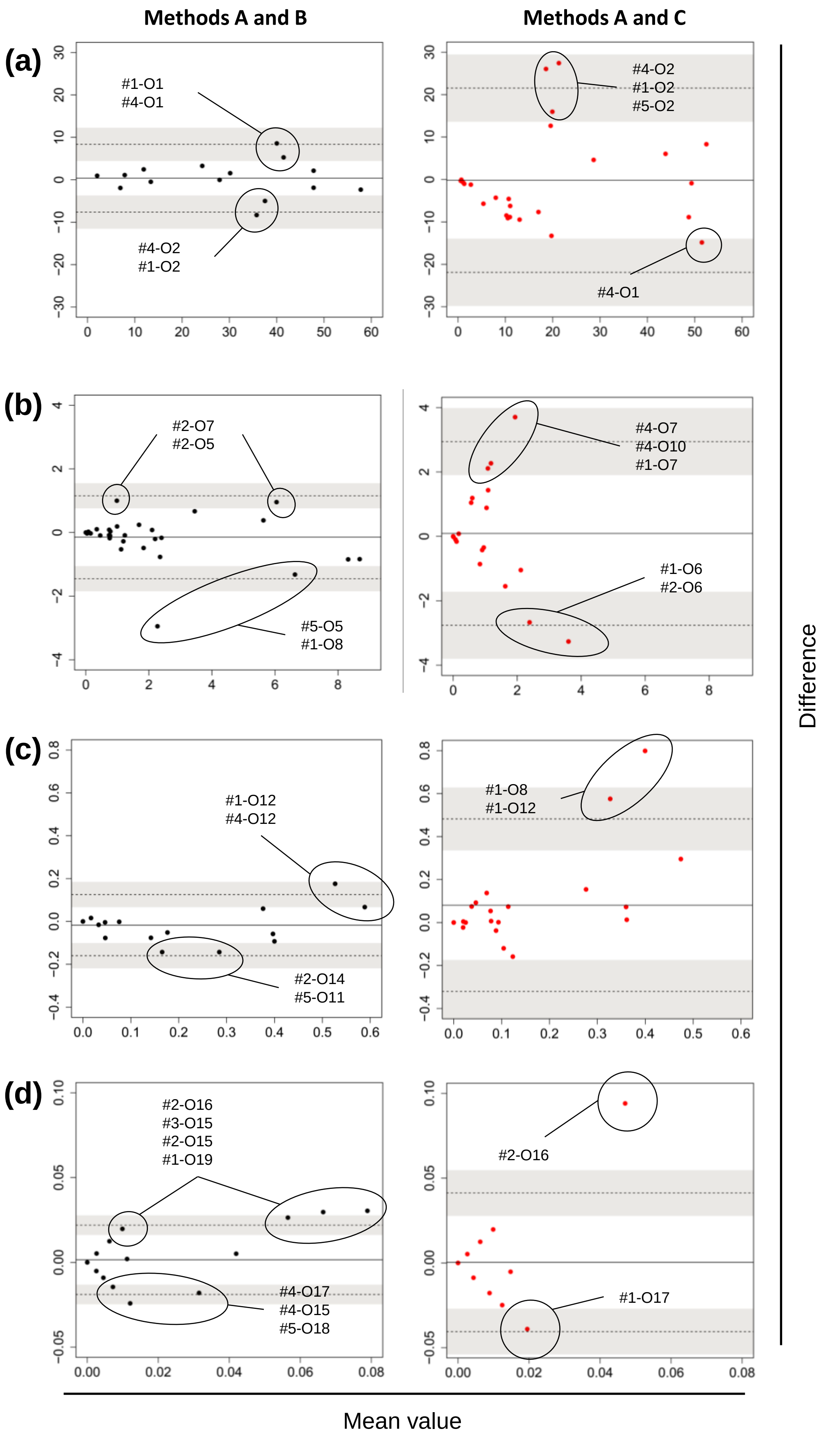

Figure S3. Research Ⅰ: Bland-Altman plots of the relative abundance of fecal bacteria at the order level obtained by each method from five adult volunteers. Comparisons between Methods A–B (left) and Methods A–C (right). The relative abundance of bacteria in each specimen was plotted. (a) Their maximum abundance in any of the specimens was ≥ 10%; (b) ≥ 1% and < 10%; (c) ≥ 0.1% and < 1%; (d) < 0.1%. Solid lines indicate the means of the differences between the two test values, dotted lines indicate the limits of agreement (the mean of the difference ± 1.96 × standard deviation), and the gray shaded area indicate the 95% confidence interval of the limits of agreement. For Methods A and B, stool samples were collected by commercial collection tubes. For Method C, urine and stool samples were applied to disposable diapers. For Methods B and C, the samples were stored at -80 ℃ for 2 months.

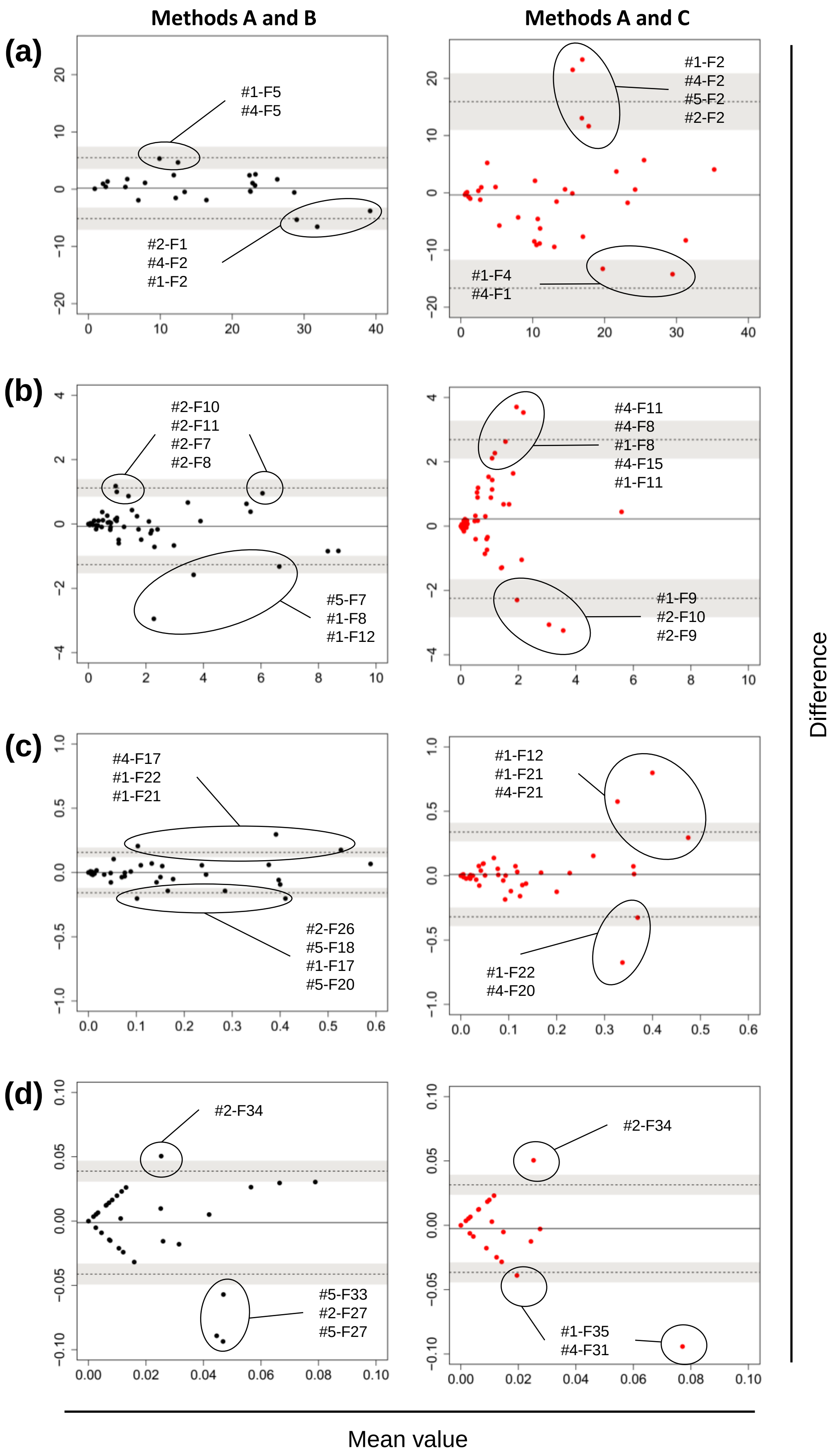

Figure S4. Research Ⅰ: Bland-Altman plots of the relative abundance of fecal bacteria at the family level obtained by each method from five adult volunteers. Comparisons between Methods A–B (left) and Methods A–C (right). The relative abundance of bacteria in each specimen was plotted. (a) Their maximum abundance in any of the specimens was ≥ 10%; (b) ≥ 1% and < 10%; (c) ≥ 0.1% and < 1%; (d) < 0.1%. Solid lines indicate the means of the differences between the two test values, dotted lines indicate the limits of agreement (the mean of the difference ± 1.96 × standard deviation), and the gray shaded area indicate the 95% confidence interval of the limits of agreement. For Methods A and B, stool samples were collected by commercial collection tubes. For Method C, urine and stool samples were applied to disposable diapers. For Methods B and C, the samples were stored at -80 ℃ for 2 months.

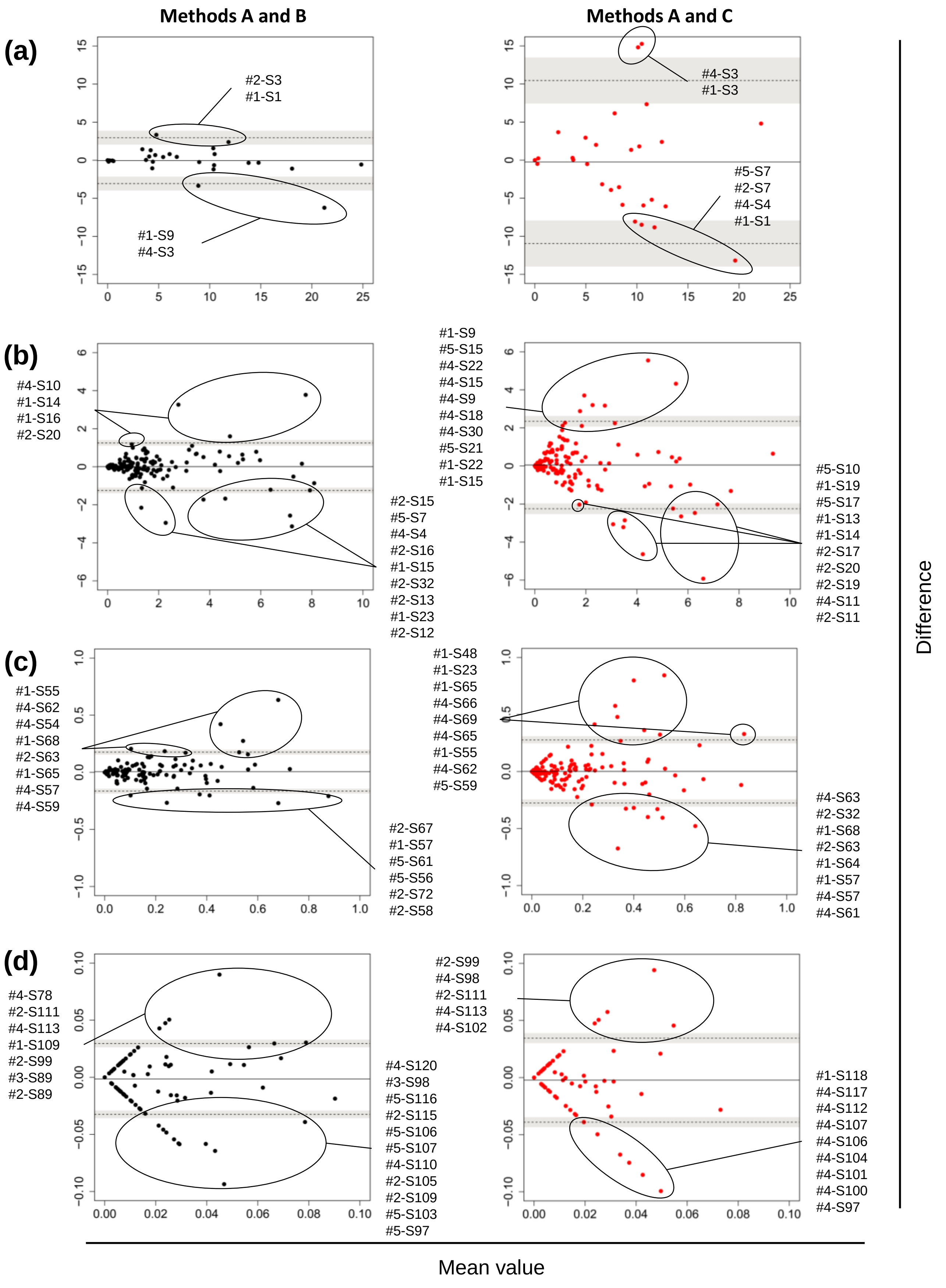

Figure S5. Research Ⅰ: Bland-Altman plots of the relative abundance of fecal bacteria at the species level obtained by each method from five adult volunteers. Comparisons between Methods A–B (left) and Methods A–C (right). The relative abundance of bacteria in each specimen was plotted. (a) Their maximum abundance in any of the specimens was ≥ 10%; (b) ≥ 1% and < 10%; (c) ≥ 0.1% and < 1%; (d) < 0.1%. Solid lines indicate the means of the differences between the two test values, dotted lines indicate the limits of agreement (the mean of the difference ± 1.96 × standard deviation), and the gray shaded area indicate the 95% confidence interval of the limits of agreement. For Methods A and B, stool samples were collected by commercial collection tubes. For Method C, urine and stool samples were applied to disposable diapers. For Methods B and C, the samples were stored at -80 ℃ for 2 months.

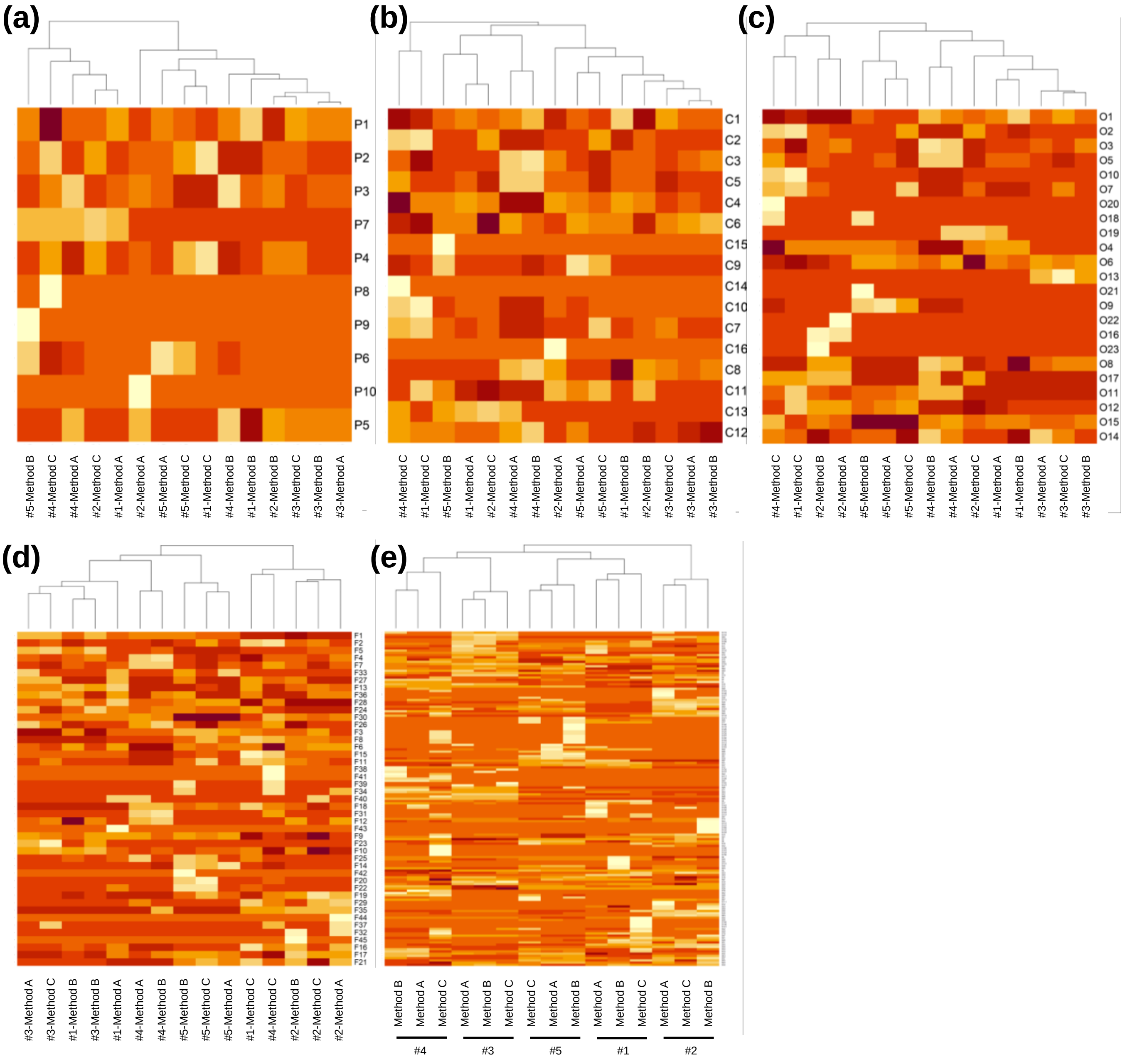

Figure S6. Research I: Heatmap of gut microbiota composition at the phylum, class, order, family, and species levels in five adult volunteers. Heatmap of log-transformed values of relative abundance at the (a) phylum, (b) class, (c) order, (d) family, and (e) species levels in the three methods of Research Ⅰ using the Ward's method for clustering.

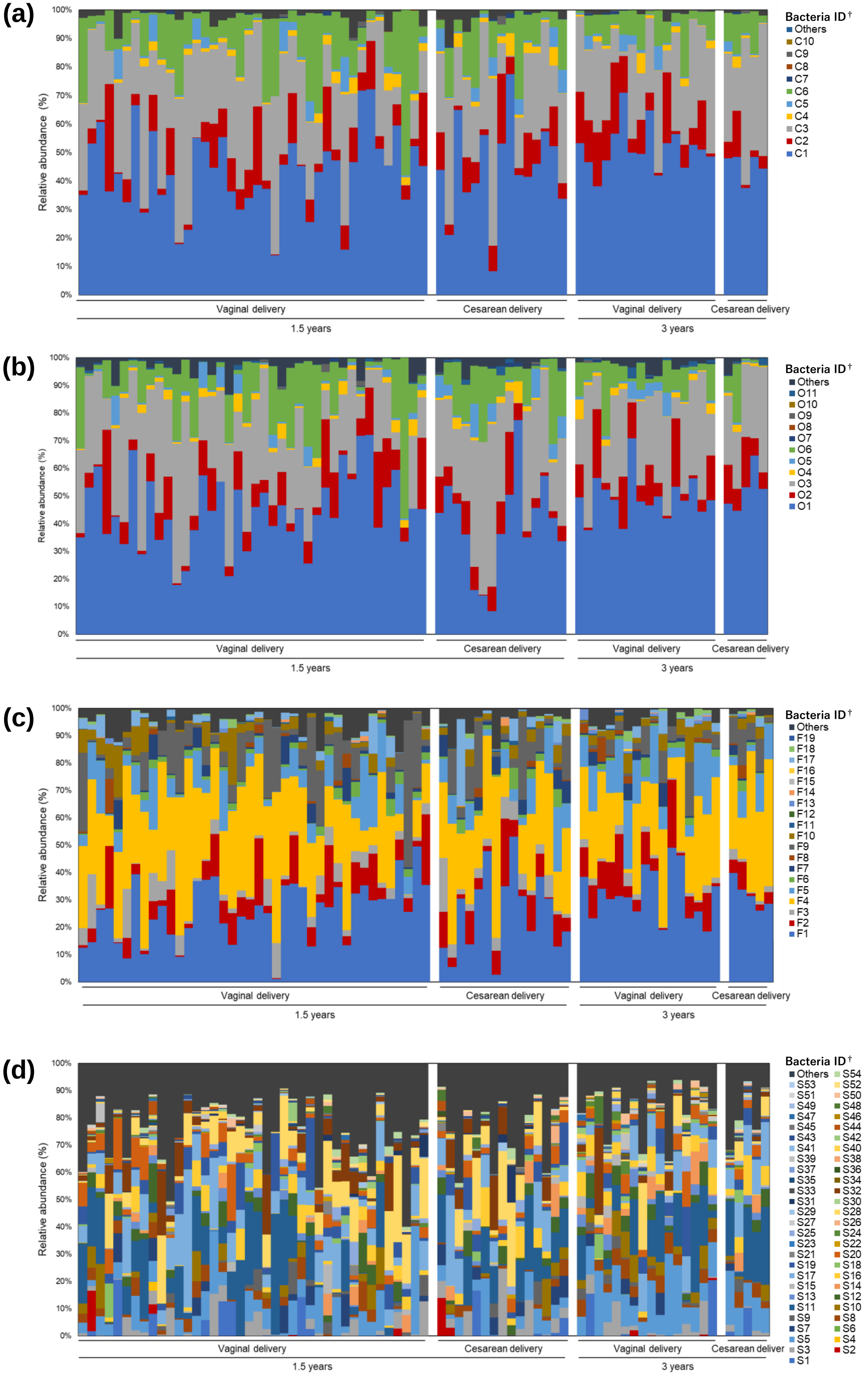

Figure S7. Research II: Fecal bacterial composition in 76 toddlers at the (a) class, (b) order, (c) family, and (d) species levels.
