# Supplementary material for: Epidemiological Studies of Children’s Gut Microbiota: Validation of Sample Collection and Storage Methods and Microbiota Analysis of Toddlers’ Feces Collected from Diapers
Source: Nutrients. 2022 Aug 12;14(16):3315. doi: 10.3390/nu14163315 (PMC9416069; doi:10.3390/nu14163315)
Supplement: Supplementary file 1 [file nutrients-14-03315-s001.zip › nutrients-1777811-supplementary/nutrients-1777811-supplementary-table.pdf]

Table S1. ID list of identified bacteria at the phylum level.

Phylum (ID)	Phylum
P1	p__Firmicutes
P2	p__Bacteroidetes
P3	p__Actinobacteria
P4	p__Proteobacteria
P5	p__Fusobacteria
P6	p__Verrucomicrobia
P7	p__TM7
P8	p__Cyanobacteria
P9	p__Lentisphaerae
P10	p__Spirochaetes
P11	__

Table S2. ID list of identified bacteria at the class level.

Class (ID)	Phylum (ID)	Class
**C1**	P1	c__Clostridia
**C2**	P2	c__Bacteroidia
**C3**	P3	c__Actinobacteria
**C4**	P1	c__Erysipelotrichi
**C5**	P3	c__Coriobacteriia
**C6**	P1	c__Bacilli
**C7**	P4	c__Betaproteobacteria
**C8**	P5	c__Fusobacteriia
**C9**	P6	c__Verrucomicrobiae
**C10**	P4	c__Alphaproteobacteria
**C11**	P4	c__Deltaproteobacteria
**C12**	P4	c__Gammaproteobacteria
**C13**	P7	c__TM7-3
**C14**	P8	c__Chloroplast
**C15**	P9	c__[Lentisphaeria]
**C16**	P10	c__[Brachyspirae]
**C17**	P11	__

Table S3. ID list of identified bacteria at the order level.

Order (ID)	Phylum (ID)	Class (ID)	Order
O1	P1	C1	o__Clostridiales
O2	P2	C2	o__Bacteroidales
O3	P3	C3	o__Bifidobacteriales
O4	P1	C4	o__Erysipelotrichales
O5	P3	C5	o__Coriobacteriales
O6	P1	C6	o__Lactobacillales
O7	P4	C7	o__Burkholderiales
O8	P5	C8	o__Fusobacteriales
O9	P6	C9	o__Verrucomicrobiales
O10	P4	C10	o__RF32
O11	P1	C6	o__Turicibacterales
O12	P4	C11	o__Desulfovibrionales
O13	P4	C12	o__Aeromonadales
O14	P4	C12	o__Enterobacteriales
O15	P3	C3	o__Actinomycetales
O16	P4	C12	o__Pasteurellales
O17	P1	C6	o__Gemellales
O18	P7	C13	o__CW040
O19	P7	C13	o__
O20	P8	C14	o__Rhodophyta
O21	P9	C15	o__Victivallales
O22	P10	C16	o__[Brachyspirales]
O23	P4	C12	o__Pseudomonadales
O24	P11	C17	__
O25	P8	C14	o__Streptophyta
O26	P1	C6	__
O27	P1	C6	o__Bacillales

Table S4. ID list of identified bacteria at the family level.

Family (ID)	Phylum (ID)	Class (ID)	Order (ID)	Family
F1	P1	C1	O1	f__Lachnospiraceae
F2	P2	C2	O2	f__Bacteroidaceae
F3	P1	C1	O1	f__Veillonellaceae
F4	P3	C3	O3	f__Bifidobacteriaceae
F5	P1	C1	O1	f__Ruminococcaceae
F6	P1	C4	O4	f__Erysipelotrichaceae
F7	P3	C5	O5	f__Coriobacteriaceae
F8	P2	C2	O2	f__Porphyromonadaceae
F9	P1	C6	O6	f__Streptococcaceae
F10	P1	C1	O1	__
F11	P4	C7	O7	f__Alcaligenaceae
F12	P5	C8	O8	f__Fusobacteriaceae
F13	P2	C2	O2	f__Rikenellaceae
F14	P6	C9	O9	f__Verrucomicrobiaceae
F15	P4	C10	O10	f__
F16	P2	C2	O2	f__[Barnesiellaceae]
F17	P1	C1	O1	f__Clostridiaceae
F18	P1	C6	O11	f__Turicibacteraceae
F19	P2	C2	O2	f__[Odoribacteraceae]
F20	P2	C2	O2	f__S24-7
F21	P4	C11	O12	f__Desulfovibrionaceae
F22	P1	C6	O6	f__
F23	P4	C12	O13	f__Succinivibrionaceae
F24	P1	C1	O1	f__[Mogibacteriaceae]
F25	P1	C1	O1	f__Eubacteriaceae
F26	P4	C12	O14	f__Enterobacteriaceae
F27	P1	C6	O6	f__Lactobacillaceae
F28	P1	C1	O1	f__
F29	P1	C6	O6	f__Enterococcaceae
F30	P3	C3	O15	f__Actinomycetaceae
F31	P1	C1	O1	f__[Tissierellaceae]
F32	P4	C12	O16	f__Pasteurellaceae
F33	P1	C1	O1	f__Christensenellaceae
F34	P2	C2	O2	f__Prevotellaceae
F35	P1	C6	O17	f__Gemellaceae
F36	P1	C6	O6	f__Carnobacteriaceae
F37	P1	C1	O1	f__Peptostreptococcaceae
F38	P2	C2	O2	f__[Paraprevotellaceae]
F39	P7	C13	O18	f__F16
F40	P7	C13	O19	f__
F41	P8	C14	O20	f__
F42	P9	C15	O21	f__Victivallaceae
F43	P1	C6	O6	f__Leuconostocaceae
F44	P10	C16	O22	f__Brachyspiraceae
F45	P4	C12	O23	f__Pseudomonadaceae
F46	P11	C17	O24	__
F47	P3	C3	O15	f__Corynebacteriaceae
F48	P3	C3	O15	f__Micrococcaceae
F49	P2	C2	O2	__
F50	P8	C14	O25	f__
F51	P1	C6	O26	__
F52	P1	C6	O27	f__Bacillaceae
F53	P1	C6	O27	f__Staphylococcaceae
F54	P1	C6	O6	__
F55	P1	C6	O6	f__Aerococcaceae
F56	P7	C13	O18	f__

Table S5. ID list of identified bacteria at the genus level.

Genus (ID)	Phylum (ID)	Class (ID)	Order (ID)	Family (ID)	Genus
G1	P2	C2	O2	F2	*g__Bacteroides*
G2	P3	C3	O3	F4	*g__Bifidobacterium*
G3	P1	C1	O1	F3	*g__Megamonas*
G4	P1	C4	O4	F6	*g__Catenibacterium*
G5	P1	C1	O1	F1	*g__Blautia*
G6	P3	C5	O5	F7	*g__Collinsella*
G7	P1	C1	O1	F1	__
G8	P1	C1	O1	F5	*g__Ruminococcus*
G9	P1	C1	O1	F5	*g__Gemmiger*
G10	P1	C1	O1	F5	*g__Faecalibacterium*
G11	P1	C1	O1	F1	*g__Roseburia*
G12	P1	C1	O1	F1	*g__Coprococcus*
G13	P2	C2	O2	F8	*g__Parabacteroides*
G14	P1	C6	O6	F9	*g__Streptococcus*
G15	P1	C1	O1	F10	__
G16	P1	C1	O1	F1	*g__Dorea*
G17	P4	C7	O7	F11	*g__Sutterella*
G18	P1	C1	O1	F1	*g__[Ruminococcus]*
G19	P5	C8	O8	F12	__
G20	P1	C1	O1	F3	*g__Dialister*
G21	P1	C4	O4	F6	*g__[Eubacterium]*
G22	P2	C2	O2	F13	*g__Alistipes*
G23	P6	C9	O9	F14	*g__Akkermansia*
G24	P4	C10	O10	F15	*g__*
G25	P1	C1	O1	F5	*g__Oscillospira*
G26	P1	C1	O1	F3	*g__Phascolarctobacterium*
G27	P1	C1	O1	F3	*g__Acidaminococcus*
G28	P1	C1	O1	F5	*g__Butyricicoccus*
G29	P1	C1	O1	F1	*g__Clostridium*
G30	P2	C2	O2	F13	*g__*
G31	P2	C2	O2	F16	*g__*
G32	P1	C1	O1	F1	*g__*
G33	P3	C5	O5	F7	*g__Eggerthella*
G34	P1	C1	O1	F3	*g__Megasphaera*
G35	P1	C6	O11	F18	*g__Turicibacter*
G36	P1	C1	O1	F1	*g__Lachnospira*
G37	P1	C1	O1	F1	*g__Anaerostipes*
G38	P1	C1	O1	F17	*g__Clostridium*
G39	P1	C4	O4	F6	*g__*
G40	P1	C1	O1	F5	__
G41	P2	C2	O2	F20	*g__*
G42	P1	C4	O4	F6	*g__Clostridium*
G43	P1	C1	O1	F3	*g__Veillonella*
G44	P4	C11	O12	F21	*g__Bilophila*
G45	P2	C2	O2	F19	*g__Butyricimonas*
G46	P1	C6	O6	F22	*g__*
G47	P2	C2	O2	F19	*g__Odoribacter*
G48	P4	C12	O13	F23	*g__Succinatimonas*
G49	P5	C8	O8	F12	*g__Fusobacterium*
G50	P1	C1	O1	F5	*g__*
G51	P1	C1	O1	F24	*g__*
G52	P1	C1	O1	F25	*g__Pseudoramibacter_Eubacterium*
G53	P1	C1	O1	F17	*g__SMB53*
G54	P4	C12	O14	F26	__
G55	P1	C4	O4	F6	*g__Coprobacillus*
G56	P1	C6	O6	F27	*g__Lactobacillus*

G57	P3	C5	O5	F7	*g__Adlercreutzia*
G58	P1	C1	O1	F28	*g__*
G59	P1	C6	O6	F29	*g__Enterococcus*
G60	P3	C3	O15	F30	*g__Actinomyces*
G61	P3	C5	O5	F7	*g__Slackia*
G62	P1	C1	O1	F17	*g__*
G63	P3	C5	O5	F7	*g__*
G64	P1	C1	O1	F31	*g__Parvimonas*
G65	P4	C12	O16	F32	*g__Haemophilus*
G66	P1	C4	O4	F6	*g__cc_115*
G67	P2	C2	O2	F13	__
G68	P1	C1	O1	F33	*g__*
G69	P1	C4	O4	F6	*g__Allobaculum*
G70	P1	C6	O6	F9	*g__Lactococcus*
G71	P4	C11	O12	F21	*g__Desulfovibrio*
G72	P2	C2	O2	F34	*g__Prevotella*
G73	P1	C1	O1	F5	*g__Anaerotruncus*
G74	P1	C6	O17	F35	__
G75	P1	C4	O4	F6	*g__Holdemania*
G76	P1	C6	O6	F36	*g__Granulicatella*
G77	P3	C5	O5	F7	__
G78	P1	C1	O1	F37	*g__Peptostreptococcus*
G79	P2	C2	O2	F38	*g__[Prevotella]*
G80	P7	C13	O18	F39	*g__*
G81	P7	C13	O19	F40	*g__*
G82	P8	C14	O20	F41	*g__*
G83	P1	C6	O6	F27	*g__Pediococcus*
G84	P3	C3	O3	F4	*g__Alloscardovia*
G85	P3	C5	O5	F7	*g__Atopobium*
G86	P1	C1	O1	F33	*g__Christensenella*
G87	P1	C4	O4	F6	*g__Bulleidia*
G88	P9	C15	O21	F42	__
G89	P1	C1	O1	F25	*g__Anaerofustis*
G90	P1	C1	O1	F31	*g__Finegoldia*
G91	P1	C6	O6	F43	*g__Weissella*
G92	P10	C16	O22	F44	*g__Brachyspira*
G93	P4	C12	O23	F45	__
G94	P11	C17	O24	F46	__
G95	P3	C3	O15	F30	*g__*
G96	P3	C3	O15	F30	*g__Varibaculum*
G97	P3	C3	O15	F47	*g__Corynebacterium*
G98	P3	C3	O15	F48	*g__Rothia*
G99	P3	C3	O3	F4	*g__Scardovia*
G100	P3	C5	O5	F7	*g__Olsenella*
G101	P2	C2	O2	F49	__
G102	P2	C2	O2	F8	*g__Dysgonomonas*
G103	P2	C2	O2	F8	*g__Porphyromonas*
G104	P2	C2	O2	F16	__
G105	P8	C14	O25	F50	*g__*
G106	P1	C6	O26	F51	__
G107	P1	C6	O27	F52	*g__Bacillus*
G108	P1	C6	O27	F53	*g__Staphylococcus*
G109	P1	C6	O17	F35	*g__*
G110	P1	C6	O6	F54	__
G111	P1	C6	O6	F55	*g__Abiotrophia*
G112	P1	C6	O6	F55	*g__Alloiococcus*
G113	P1	C6	O6	F43	*g__Leuconostoc*
G114	P1	C1	O1	F17	__

G115	P1	C1	O1	F1	*g__Epulopiscium*
G116	P1	C1	O1	F1	*g__Lactonifactor*
G117	P1	C1	O1	F1	*g__Robinsoniella*
G118	P1	C1	O1	F1	*g__Ruminococcus*
G119	P1	C1	O1	F37	__
G120	P1	C1	O1	F3	__
G121	P1	C1	O1	F3	*g__Mitsuokella*
G122	P1	C1	O1	F24	*g__Mogibacterium*
G123	P1	C1	O1	F31	*g__Anaerococcus*
G124	P1	C1	O1	F31	*g__Peptoniphilus*
G125	P1	C1	O1	F31	*g__WAL_1855D*
G126	P1	C4	O4	F6	__
G127	P4	C11	O12	F21	*g__*
G128	P4	C12	O14	F26	*g__Citrobacter*
G129	P4	C12	O14	F26	*g__Enterobacter*
G130	P4	C12	O14	F26	*g__Klebsiella*
G131	P4	C12	O14	F26	*g__Morganella*
G132	P4	C12	O14	F26	*g__Proteus*
G133	P4	C12	O14	F26	*g__Trabulsiella*
G134	P4	C12	O16	F32	__
G135	P4	C12	O16	F32	*g__Actinobacillus*
G136	P4	C12	O23	F45	*g__Pseudomonas*
G137	P7	C13	O18	F56	*g__*

Table S6. ID list of identified bacteria at the species level.

Species (ID)	Phylum (ID)	Class (ID)	Order (ID)	Family (ID)	Genus (ID)	Species
S1	P3	C3	O3	F4	G2	s__adolescentis
S2	P1	C1	O1	F3	G3	s__
S3	P2	C2	O2	F2	G1	s__
S4	P1	C4	O4	F6	G4	s__
S5	P1	C1	O1	F1	G5	s__
S6	P2	C2	O2	F2	G1	s__plebeius
S7	P3	C5	O5	F7	G6	s__aerofaciens
S8	P1	C1	O1	F1	G7	__
S9	P2	C2	O2	F2	G1	s__ovatus
S10	P1	C1	O1	F5	G9	s__formicilis
S11	P3	C3	O3	F4	G2	s__
S12	P1	C1	O1	F5	G10	s__prausnitzii
S13	P1	C1	O1	F1	G11	__
S14	P1	C1	O1	F5	G8	s__bromii
S15	P2	C2	O2	F2	G1	s__uniformis
S16	P1	C1	O1	F1	G12	s__
S17	P3	C3	O3	F4	G2	s__longum
S18	P2	C2	O2	F8	G13	s__distasonis
S19	P1	C6	O6	F9	G14	s__
S20	P1	C1	O1	F10	G15	__
S21	P2	C2	O2	F2	G1	__
S22	P4	C7	O7	F11	G17	s__
S23	P5	C8	O8	F12	G19	__
S24	P1	C1	O1	F3	G20	s__
S25	P1	C1	O1	F1	G16	s__
S26	P1	C4	O4	F6	G21	s__biforme
S27	P1	C1	O1	F1	G16	s__longicatena
S28	P1	C1	O1	F1	G18	s__gnavus
S29	P6	C9	O9	F14	G23	s__muciniphila
S30	P4	C10	O10	F15	G24	s__
S31	P1	C1	O1	F1	G5	s__producta
S32	P3	C3	O3	F4	G2	s__breve
S33	P1	C1	O1	F5	G25	s__
S34	P1	C1	O1	F1	G12	s__eutactus
S35	P1	C1	O1	F3	G26	s__
S36	P1	C1	O1	F3	G27	s__
S37	P1	C1	O1	F5	G28	s__pullicaecorum
S38	P2	C2	O2	F13	G30	s__
S39	P1	C1	O1	F1	G5	s__obeum
S40	P1	C1	O1	F1	G11	s__faecis
S41	P2	C2	O2	F16	G31	s__
S42	P1	C1	O1	F1	G32	s__
S43	P3	C5	O5	F7	G33	s__lenta
S44	P1	C1	O1	F3	G34	s__
S45	P1	C1	O1	F5	G8	s__
S46	P2	C2	O2	F13	G22	s__putredinis
S47	P2	C2	O2	F8	G13	s__
S48	P2	C2	O2	F2	G1	s__caccae
S49	P2	C2	O2	F13	G22	s__massiliensis
S50	P1	C6	O11	F18	G35	s__
S51	P1	C1	O1	F1	G36	s__
S52	P1	C1	O1	F1	G29	s__citroniae
S53	P1	C1	O1	F1	G37	s__
S54	P1	C4	O4	F6	G21	s__dolichum
S55	P1	C1	O1	F1	G18	s__
S56	P1	C1	O1	F5	G8	__

**S57**	P1	C1	O1	F17	G38	s__
**S58**	P1	C4	O4	F6	G39	s__
**S59**	P1	C1	O1	F5	G40	__
**S60**	P1	C1	O1	F1	G18	s__torques
**S61**	P2	C2	O2	F20	G41	s__
**S62**	P1	C1	O1	F1	G29	__
**S63**	P1	C1	O1	F1	G16	s__formicigenerans
**S64**	P1	C4	O4	F6	G42	s__ramosum
**S65**	P4	C11	O12	F21	G44	s__
**S66**	P2	C2	O2	F19	G45	s__
**S67**	P1	C1	O1	F1	G12	s__catus
**S68**	P1	C6	O6	F22	G46	s__
**S69**	P2	C2	O2	F19	G47	s__
**S70**	P4	C12	O13	F23	G48	s__hippei
**S71**	P5	C8	O8	F12	G49	s__
**S72**	P1	C1	O1	F3	G43	s__parvula
**S73**	P2	C2	O2	F2	G1	s__fragilis
**S74**	P1	C1	O1	F5	G50	s__
**S75**	P1	C6	O6	F9	G14	__
**S76**	P1	C1	O1	F24	G51	s__
**S77**	P1	C1	O1	F25	G52	s__
**S78**	P1	C1	O1	F17	G53	s__
**S79**	P1	C1	O1	F1	G16	__
**S80**	P4	C12	O14	F26	G54	__
**S81**	P1	C4	O4	F6	G55	s__
**S82**	P1	C1	O1	F1	G29	s__aldenense
**S83**	P1	C1	O1	F3	G43	__
**S84**	P2	C2	O2	F13	G22	s__onderdonkii
**S85**	P1	C1	O1	F1	G29	s__symbiosum
**S86**	P3	C5	O5	F7	G57	s__
**S87**	P1	C1	O1	F28	G58	s__
**S88**	P1	C6	O6	F29	G59	s__
**S89**	P3	C3	O15	F30	G60	s__
**S90**	P3	C5	O5	F7	G61	s__
**S91**	P1	C1	O1	F1	G18	__
**S92**	P1	C1	O1	F17	G62	s__
**S93**	P1	C1	O1	F5	G25	__
**S94**	P3	C5	O5	F7	G63	s__
**S95**	P1	C1	O1	F31	G64	s__
**S96**	P1	C4	O4	F6	G42	s__spiroforme
**S97**	P1	C6	O6	F27	G56	s__
**S98**	P1	C1	O1	F1	G29	s__lavalense
**S99**	P4	C12	O16	F32	G65	s__parainfluenzae
**S100**	P1	C6	O6	F27	G56	__
**S101**	P1	C4	O4	F6	G66	s__
**S102**	P2	C2	O2	F13	G67	__
**S103**	P1	C1	O1	F33	G68	s__
**S104**	P1	C4	O4	F6	G69	s__
**S105**	P1	C6	O6	F9	G70	s__
**S106**	P2	C2	O2	F2	G1	s__acidifaciens
**S107**	P4	C11	O12	F21	G71	s__C21_c20
**S108**	P2	C2	O2	F13	G22	s__finegoldii
**S109**	P1	C1	O1	F3	G43	s__dispar
**S110**	P1	C1	O1	F1	G29	s__hathewayi
**S111**	P2	C2	O2	F34	G72	s__copri
**S112**	P3	C3	O3	F4	G2	s__pseudolongum
**S113**	P1	C1	O1	F17	G38	s__celatum
**S114**	P1	C6	O6	F9	G14	s__anginosus

**S115**	P1	C6	O6	F27	G56	s__plantarum
**S116**	P1	C1	O1	F5	G73	s__
**S117**	P2	C2	O2	F13	G22	s__indistinctus
**S118**	P1	C6	O17	F35	G74	__
**S119**	P1	C4	O4	F6	G75	s__
**S120**	P1	C6	O6	F36	G76	s__
**S121**	P3	C5	O5	F7	G77	__
**S122**	P1	C1	O1	F37	G78	s__
**S123**	P1	C6	O6	F27	G56	s__zeae
**S124**	P2	C2	O2	F38	G79	s__
**S125**	P7	C13	O18	F39	G80	s__
**S126**	P1	C1	O1	F17	G38	__
**S127**	P1	C6	O6	F27	G56	s__salivarius
**S128**	P2	C2	O2	F34	G72	s__
**S129**	P7	C13	O19	F40	G81	s__
**S130**	P8	C14	O20	F41	G82	s__
**S131**	P1	C1	O1	F5	G8	s__callidus
**S132**	P1	C6	O6	F27	G83	s__acidilactici
**S133**	P4	C11	O12	F21	G71	s__
**S134**	P3	C3	O3	F4	G84	s__
**S135**	P3	C5	O5	F7	G85	s__
**S136**	P1	C1	O1	F33	G86	s__
**S137**	P1	C4	O4	F6	G87	s__moorei
**S138**	P1	C4	O4	F6	G55	s__cateniformis
**S139**	P1	C6	O6	F29	G59	s__casseliflavus
**S140**	P9	C15	O21	F42	G88	__
**S141**	P1	C6	O6	F9	G70	s__garvieae
**S142**	P1	C1	O1	F25	G89	s__
**S143**	P1	C1	O1	F31	G90	s__
**S144**	P1	C6	O6	F43	G91	__
**S145**	P1	C1	O1	F37	G78	s__anaerobius
**S146**	P1	C6	O6	F27	G56	s__helveticus
**S147**	P1	C1	O1	F17	G38	s__perfringens
**S148**	P10	C16	O22	F44	G92	s__
**S149**	P4	C12	O23	F45	G93	__
**S150**	P11	C17	O24	F46	G94	__
**S151**	P3	C3	O15	F30	G95	s__
**S152**	P3	C3	O15	F30	G96	s__
**S153**	P3	C3	O15	F47	G97	s__
**S154**	P3	C3	O15	F47	G97	s__simulans
**S155**	P3	C3	O15	F48	G98	s__dentocariosa
**S156**	P3	C3	O15	F48	G98	s__mucilaginosa
**S157**	P3	C3	O3	F4	G2	__
**S158**	P3	C3	O3	F4	G2	s__animalis
**S159**	P3	C3	O3	F4	G2	s__bifidum
**S160**	P3	C3	O3	F4	G99	s__
**S161**	P3	C5	O5	F7	G85	s__rimae
**S162**	P3	C5	O5	F7	G6	__
**S163**	P3	C5	O5	F7	G6	s__
**S164**	P3	C5	O5	F7	G33	s__
**S165**	P3	C5	O5	F7	G100	s__uli
**S166**	P2	C2	O2	F49	G101	__
**S167**	P2	C2	O2	F2	G1	s__eggerthii
**S168**	P2	C2	O2	F8	G102	s__
**S169**	P2	C2	O2	F8	G102	s__gadei
**S170**	P2	C2	O2	F8	G13	__
**S171**	P2	C2	O2	F8	G103	s__
**S172**	P2	C2	O2	F16	G104	__

S173	P8	C14	O25	F50	G105	s__
S174	P1	C6	O26	F51	G106	__
S175	P1	C6	O27	F52	G107	__
S176	P1	C6	O27	F52	G107	s__
S177	P1	C6	O27	F53	G108	__
S178	P1	C6	O17	F35	G109	s__
S179	P1	C6	O6	F54	G110	__
S180	P1	C6	O6	F55	G111	s__
S181	P1	C6	O6	F55	G112	s__
S182	P1	C6	O6	F29	G59	__
S183	P1	C6	O6	F27	G56	s__delbrueckii
S184	P1	C6	O6	F27	G56	s__mucosae
S185	P1	C6	O6	F27	G56	s__reuteri
S186	P1	C6	O6	F27	G83	s__
S187	P1	C6	O6	F43	G113	s__
S188	P1	C6	O6	F9	G14	s__agalactiae
S189	P1	C6	O6	F9	G14	s__alactolyticus
S190	P1	C6	O6	F9	G14	s__luteciae
S191	P1	C6	O6	F9	G14	s__sobrinus
S192	P1	C1	O1	F17	G114	__
S193	P1	C1	O1	F17	G38	s__butyricum
S194	P1	C1	O1	F17	G38	s__neonatale
S195	P1	C1	O1	F17	G38	s__paraputrificum
S196	P1	C1	O1	F1	G5	__
S197	P1	C1	O1	F1	G29	s__colinum
S198	P1	C1	O1	F1	G29	s__piliforme
S199	P1	C1	O1	F1	G115	s__
S200	P1	C1	O1	F1	G116	s__longoviformis
S201	P1	C1	O1	F1	G117	s__peoriensis
S202	P1	C1	O1	F1	G11	s__
S203	P1	C1	O1	F1	G118	s__gauvreauii
S204	P1	C1	O1	F37	G119	__
S205	P1	C1	O1	F5	G10	__
S206	P1	C1	O1	F3	G120	__
S207	P1	C1	O1	F3	G121	__
S208	P1	C1	O1	F3	G121	s__
S209	P1	C1	O1	F3	G43	s__
S210	P1	C1	O1	F24	G122	s__
S211	P1	C1	O1	F31	G123	s__
S212	P1	C1	O1	F31	G124	s__
S213	P1	C1	O1	F31	G125	s__
S214	P1	C4	O4	F6	G126	__
S215	P1	C4	O4	F6	G42	s__saccharogumia
S216	P4	C11	O12	F21	G127	s__
S217	P4	C12	O14	F26	G128	__
S218	P4	C12	O14	F26	G128	s__
S219	P4	C12	O14	F26	G129	__
S220	P4	C12	O14	F26	G130	__
S221	P4	C12	O14	F26	G131	s__morganii
S222	P4	C12	O14	F26	G132	s__
S223	P4	C12	O14	F26	G133	__
S224	P4	C12	O16	F32	G134	__
S225	P4	C12	O16	F32	G135	s__
S226	P4	C12	O16	F32	G135	s__parahaemolyticus
S227	P4	C12	O23	F45	G136	s__fragi
S228	P7	C13	O18	F56	G137	s__

Table S7. **Research I: Summary of bacteria detection and** Bland-Altman **plotting results (Methods A-B (a) and Methods A-C (b))** at class level.

(a)

Max relative abundance	Bacteria ID†	Number of specimens with the bacteria detection (/5)		Number of specimens with the bacteria abundance out of LOA	
		Method A	Method B	SD€	CI∫
≥10%	C1	5	5	1	2
	C2	5	5	1	2
	C3	5	5	0	0
≥1%, <10%	C4	5	5	0	0
	C5	5	5	0	2
	C6	5	5	0	0
	C7	5	5	0	1
	C8	4	4	1	1
	C9	2	2	0	0
	C10	2	2	0	0
<1%	C11	5	5	1	1
	C12	5	5	1	2
	C13	2	1	0	0
	C15	0	1	0	0
	C16	1	0	0	0

(b)

Max relative abundance	Bacteria ID†	Number of specimens with the bacteria detection (/5)		Number of specimens with the bacteria abundance out of LOA	
		Method A	Method C	SD€	CI∫
≥10%	C1	5	5	0	1
	C2	5	5	2	3
	C3	5	5	0	0
	C4	5	5	0	0
	C5	5	5	0	0
≥1%, <10%	C6	5	5	1	2
	C7	5	5	1	2
	C9	2	2	0	0
	C10	2	2	0	1
<1%	C8	4	1	1	1
	C11	5	4	1	1
	C12	5	5	0	0
	C13	2	2	0	0
	C14	0	1	0	0
	C16	1	0	0	0

†See Table S2 for bacterial taxonomy name corresponding to each ID. €SD, LOA defined as mean ± 1.96 × standard deviation of differences between two measurements. ∫CI, LOA defined as lower or upper limit of 95% confidence interval of upper or lower limit, respectively, of SD. LOA, limit of agreement.

Table S8. **Research I: Summary of bacteria detection and** Bland-Altman **plotting results (Methods A-B (a) and Methods A-C (b)) at order level.**

(a)

Maximum relative abundance	Bacteria ID[†]	Number of specimens with the bacteria detection (/5)		Number of specimens with the bacteria abundance out of the LOA	
		Method A	Method B	SD[€]	CI[ʃ]
≥10%	O1	5	5	1	2
	O2	5	5	1	2
	O3	5	5	0	0
≥1%, <10%	O4	5	5	0	0
	O5	5	5	0	2
	O6	5	5	0	0
	O7	5	5	0	1
	O8	4	4	1	1
	O9	2	2	0	0
	O10	2	2	0	0
≥0.1%, <1%	O11	3	3	0	1
	O12	5	5	1	2
	O13	1	1	0	0
	O14	5	5	0	1
<0.1%	O15	4	4	2	3
	O16	1	1	1	1
	O17	1	2	0	1
	O18	0	1	1	1
	O19	2	0	0	1
	O21	0	1	0	0
	O22	1	0	0	0
	O23	0	1	0	0

(b)

Maximum relative abundance	Bacteria ID[†]	Number of specimens with the bacteria detection (/5)		Number of specimens with the bacteria abundance out of the LOA	
		Method A	Method C	SD[€]	CI[ʃ]
≥10%	O1	5	5	0	1
	O2	5	5	2	3
	O3	5	5	0	0
	O4	5	5	0	0
	O5	5	5	0	0
≥1%, <10%	O6	5	5	1	2
	O7	5	5	1	2
	O9	2	2	0	0
	O10	2	2	0	1
	O11	3	4	0	0
≥0.1%, <1%	O8	4	1	1	1
	O12	5	4	1	1
	O13	1	1	0	0
	O14	5	5	0	0
	O15	4	4	0	0
<0.1%	O16	1	0	1	1
	O17	1	3	0	1
	O18	0	1	0	0
	O19	2	1	0	0
	O20	0	1	0	0
	O22	1	0	0	0

[†]See Table S3 for bacterial taxonomy name corresponding to each ID. [€]SD, LOA defined as mean ± 1.96 × standard deviation of differences between two measurements. [ʃ]CI, LOA defined as lower or upper limit of 95% confidence interval of upper or lower limit, respectively, of SD. LOA, limit of agreement.

Table S9. **Research I: Summary of bacteria detection and Bland-Altman plotting results (Methods A-B (a) and Methods A-C (b)) at family level.**

(a)

Maximum relative abundance	Bacteria ID†	Number of specimens with the bacteria detection (/5)		Number of specimens with the bacteria abundance out of the LOA	
		**Method A**	**Method B**	**SD€**	**CI∫**
≥10%	F1	5	5	0	1
	F2	5	5	2	2
	F3	5	5	0	0
	F4	5	5	0	0
	F5	5	5	0	2
≥1%, <10%	F6	5	5	0	0
	F7	5	5	1	2
	F8	5	5	1	2
	F9	5	5	0	0
	F10	5	5	1	1
	F11	5	5	0	1
	F12	4	4	1	1
	F13	5	5	0	0
	F14	2	2	0	0
	F15	2	2	0	0
	F16	4	4	0	0
	F19	4	4	0	0
≥0.1%, <1%	F17	4	4	2	2
	F18	3	3	0	1
	F20	0	1	1	1
	F21	5	5	1	1
	F22	1	1	1	1
	F23	1	1	0	0
	F24	4	4	0	0
	F25	2	2	0	0
	F26	5	5	0	1
	F28	4	4	0	0
	F29	2	0	0	0
<0.1%	F27	2	2	2	2
	F30	4	4	0	0
	F31	1	1	0	0
	F32	1	1	0	0
	F33	3	1	1	1
	F34	1	1	1	1
	F35	1	2	0	0
	F36	3	3	0	0
	F37	1	0	0	0
	F39	0	1	0	0
	F40	2	0	0	0
	F42	0	1	0	0
	F43	1	0	0	0
	F44	1	0	0	0
	F45	0	1	0	0

(b)

Maximum relative abundance	Bacteria ID†	Number of specimens with the bacteria detection (/5)		Number of specimens with the bacteria abundance out of the LOA	
		**Method A**	**Method C**	**SD€**	**CI∫**
≥10%	F1	5	5	0	1
	F2	5	5	2	4
	F3	5	5	0	0
	F4	5	5	0	1
	F5	5	5	0	0
	F6	5	5	0	0
	F7	5	5	0	0
≥1%, <10%	F8	5	5	1	2
	F9	5	5	2	2
	F10	5	5	1	1
	F11	5	5	1	2
	F13	5	5	0	0
	F14	2	2	0	0
	F15	2	2	0	1
	F16	4	3	0	0
	F17	4	4	0	0
	F18	3	4	0	0
	F19	4	4	0	0
≥0.1%, <1%	F12	4	1	1	1
	F20	0	2	1	1
	F21	5	4	1	2
	F22	1	2	1	1
	F23	1	1	0	0
	F24	4	3	0	0
	F25	2	2	0	0
	F26	5	5	0	0
	F27	2	3	0	0
	F28	4	3	0	0
	F29	2	3	0	0
	F30	4	4	0	0
<0.1%	F31	1	1	1	1
	F32	1	0	0	0
	F33	3	0	0	0
	F34	1	1	1	1
	F35	1	3	1	1
	F36	3	3	0	0
	F37	1	2	0	0
	F38	0	1	0	0
	F39	0	1	0	0
	F40	2	1	0	0
	F41	0	1	0	0
	F43	1	0	0	0
	F44	1	0	0	0

†See Table S4 for bacterial taxonomy name corresponding to each ID. €SD, LOA defined as mean ± 1.96 × standard deviation of differences between two measurements. ∫CI, LOA defined as lower or upper limit of 95% confidence interval of upper or lower limit, respectively, of SD. LOA, limit of agreement.

Table S10. **Research I: Summary of bacteria detection and** Bland-Altman **plotting results (Methods A-B (a) and Methods A-C (b)) at species level.**

(a)

Maximum relative abundance	Bacteria ID†	Number of specimens with the bacteria detection (/5)		Number of specimens with the bacteria abundance out of the LOA	
		Method A	Method B	SD€	CI∫
≥10%	S1	3	4	0	1
	S2	2	2	0	0
	S3	5	5	2	2
	S5	5	5	0	0
	S6	1	2	0	0
	S8	5	5	0	0
	S9	5	5	1	1
≥1%, <10%	S4	1	1	0	1
	S7	4	4	0	1
	S10	4	4	1	1
	S11	4	2	0	0
	S12	3	4	1	1
	S13	3	3	1	1
	S14	2	3	1	1
	S15	5	5	1	2
	S16	5	5	2	2
	S17	5	4	0	0
	S18	5	5	0	0
	S19	5	5	0	0
	S20	5	5	0	1
	S21	3	3	0	0
	S22	5	5	0	0
	S23	2	2	1	1
	S24	3	4	0	0
	S25	3	3	0	0
	S26	1	1	0	0
	S27	3	3	0	0
	S28	5	5	0	0
	S29	2	2	0	0
	S30	2	2	0	0
	S31	4	4	0	0
	S32	1	1	1	1
	S33	5	5	0	0
	S34	1	1	0	0
	S35	2	3	0	0
	S36	4	4	0	0
	S37	5	4	0	0
	S38	4	3	0	0
	S39	3	3	0	0
	S40	3	3	0	0
	S41	4	4	0	0
	S42	5	5	0	0
	S44	1	2	0	0
	S45	5	5	0	0
	S46	2	3	0	0
	S47	4	5	0	0
	S48	3	3	0	0
	S49	2	2	0	0
	S51	3	4	0	0
	S52	4	3	0	0
	S53	3	4	0	0
≥0.1%, <1%	S43	5	5	0	0
	S50	3	3	0	0
	S54	4	4	1	1
	S55	5	5	1	1
	S56	1	1	1	1
	S57	4	4	1	2
	S58	2	3	1	1
	S59	5	5	0	1
	S60	3	3	0	0
	S61	0	1	1	1
	S62	1	1	1	1
	S63	3	3	1	1
	S64	3	3	0	0
	S65	5	5	0	1
	S66	2	2	0	0
	S67	2	2	1	1
	S68	1	1	1	1
	S69	4	4	0	0
	S70	1	1	0	0
	S71	2	2	0	0
	S72	1	1	1	1
	S73	3	3	0	0
	S74	4	4	0	0
	S75	3	5	0	0
	S76	4	4	0	0
	S77	1	2	0	0
	S79	1	1	0	0
	S80	5	5	0	0
	S81	5	3	0	0
	S82	3	2	0	0
	S83	1	1	0	0
	S84	2	2	0	0
	S85	2	2	0	0
	S86	1	1	0	0
	S87	4	4	0	0
	S88	2	0	0	0
	S93	1	1	0	0
	S96	3	3	0	0
<0.1%	S78	1	0	1	1
	S89	4	4	1	2
	S90	1	1	0	0
	S91	1	1	0	0
	S92	0	1	0	0
	S94	4	4	0	0
	S95	1	1	0	0
	S97	1	1	1	1
	S98	2	3	1	1
	S99	1	1	1	1
	S101	3	1	0	0
	S102	2	3	0	0
	S103	2	1	1	1
	S104	0	1	0	0
	S105	2	2	1	1
	S106	0	1	1	1
	S107	0	1	1	1
	S108	1	1	0	0
	S109	2	2	2	2
	S110	2	2	1	1
	S111	1	0	1	1
	S112	0	1	0	0
	S113	3	1	1	1
	S114	1	0	0	0
	S115	0	1	1	1
	S116	0	1	1	1
	S117	0	1	0	0
	S118	1	2	0	0
	S119	2	2	0	0
	S120	3	3	0	1
	S121	0	1	0	0
	S122	1	0	0	0
	S123	0	1	0	0
	S125	0	1	0	0
	S126	0	1	0	0
	S127	1	0	0	0
	S128	0	1	0	0
	S129	2	0	0	0
	S131	0	1	0	0
	S132	0	1	0	0
	S133	0	1	0	0
	S134	1	1	0	0
	S135	0	1	0	0
	S136	2	0	0	0
	S137	0	2	0	0
	S138	1	0	0	0
	S140	0	1	0	0
	S142	2	0	0	0
	S144	1	0	0	0
	S147	1	0	0	0
	S148	1	0	0	0
	S149	0	1	0	0

(b)

Maximum relative abundance	ID[†]	Number of specimens with the bacteria detection (/5)		Number of specimens with the bacteria abundance out of the LOA	
		**Method A**	**Method C**	**SD[†]**	**CI[∫]**
≥10%	S1	3	3	1	1
	S2	2	2	0	0
	S3	5	5	2	2
	S4	1	2	0	1
	S5	5	5	0	0
	S6	1	1	0	0
	S7	4	4	0	2
	S8	5	5	0	0
≥1%, <10%	S9	5	5	2	2
	S10	4	4	0	1
	S11	4	4	2	2
	S12	3	3	0	0
	S13	3	3	1	1
	S14	2	2	1	1
	S15	5	5	2	3
	S16	5	5	0	0
	S17	5	5	1	2
	S18	5	5	1	1
	S19	5	5	1	2
	S20	5	5	1	1
	S21	3	4	0	1
	S22	5	5	1	2
	S24	3	3	0	0
	S25	3	3	0	0
	S26	1	1	0	0
	S27	3	3	0	0
	S28	5	5	0	0
	S29	2	2	0	0
	S30	2	2	0	1
	S31	4	4	0	0
	S33	5	5	0	0
	S34	1	2	0	0
	S35	2	2	0	0
	S36	4	4	0	0
	S37	5	4	0	0
	S38	4	2	0	0
	S39	3	3	0	0
	S40	3	3	0	0
	S41	4	3	0	0
	S42	5	5	0	0
	S43	5	5	0	0
	S44	1	1	0	0
	S45	5	5	0	0
	S46	2	3	0	0
	S47	4	4	0	0
	S49	2	2	0	0
	S50	3	4	0	0
	S51	3	4	0	0
	S54	4	4	0	0
≥0.1%, <1%	S23	2	0	1	1
	S32	1	2	1	1
	S48	3	3	1	1
	S52	4	3	0	0
	S53	3	3	0	0
	S55	5	5	1	1
	S56	1	2	0	0
	S57	4	4	2	2
	S58	2	2	0	0
	S59	5	5	0	1
	S60	3	3	0	0
	S61	0	2	1	1
	S62	1	1	1	1
	S63	3	3	2	2
	S64	3	3	1	1
	S65	5	4	2	2
	S66	2	2	1	1
	S67	2	3	0	0
	S68	1	2	1	1
	S69	4	4	1	1
	S70	1	1	0	0
	S71	2	1	0	0
	S72	1	0	0	0
	S73	3	3	0	0
	S74	4	3	0	0
	S75	3	4	0	0
	S76	4	3	0	0
	S77	1	2	0	0
	S78	1	1	0	0
	S79	1	2	0	0
	S80	5	5	0	0
	S81	5	4	0	0
	S82	3	2	0	0
	S83	1	1	0	0
	S84	2	2	0	0
	S85	2	2	0	0
	S86	1	2	0	0
	S87	4	3	0	0
	S88	2	2	0	0
	S89	4	4	0	0
	S90	1	2	0	0
	S91	1	1	0	0
	S92	0	1	0	0
	S93	1	0	0	0
	S94	4	4	0	0
	S95	1	1	0	0
	S96	3	3	0	0
<0.1%	S97	1	2	1	1
	S98	2	1	1	1
	S99	1	0	1	1
	S100	0	1	1	1
	S101	3	4	1	1
	S102	2	3	1	1
	S103	2	0	0	0
	S104	0	1	1	1
	S105	2	1	0	0
	S106	0	1	1	1
	S107	0	1	1	1
	S108	1	1	0	0
	S109	2	1	0	0
	S110	2	0	0	0
	S111	1	0	1	1
	S112	0	1	1	1
	S113	3	1	1	1
	S114	1	2	0	0
	S117	0	1	0	1
	S118	1	3	0	1
	S119	2	1	0	0
	S120	3	3	0	0
	S121	0	1	0	0
	S122	1	1	0	0
	S124	0	1	0	0
	S125	0	1	0	0
	S127	1	1	0	0
	S128	0	1	0	0
	S129	2	1	0	0
	S130	0	1	0	0
	S134	1	1	0	0
	S135	0	1	0	0
	S136	2	0	0	0
	S138	1	0	0	0
	S139	0	1	0	0
	S141	0	1	0	0
	S142	2	0	0	0
	S143	0	1	0	0
	S144	1	0	0	0
	S145	0	1	0	0
	S146	0	1	0	0
	S147	1	0	0	0
	S148	1	0	0	0

[†]See Table S6 for bacterial taxonomy name corresponding to each ID. [€]SD, LOA defined as mean ± 1.96 × standard deviation of differences between two measurements. [∫]CI, LOA defined as lower or upper limit of 95% confidence interval of upper or lower limit, respectively, of SD. LOA, limit of agreement.
